# Association between triglyceride-glucose index and chronic kidney disease: results from NHANES 1999–2020

**DOI:** 10.1007/s11255-024-04103-8

**Published:** 2024-06-10

**Authors:** Xiaowan Li, Lanyu Wang, Hongyi Zhou, Hongyang Xu

**Affiliations:** 1 Department of Critical Care Medicine, The Affiliated Wuxi People’s Hospital of Nanjing Medical University, Wuxi People’s Hospital, Wuxi Medical Center, Nanjing Medical University, Wuxi, China; 2 Department of Urology, The Affiliated Wuxi People’s Hospital of Nanjing Medical University, Wuxi People’s Hospital, Wuxi Medical Center, Nanjing Medical University, Wuxi, China

**Keywords:** Triglyceride-glucose index, Chronic kidney disease, Albuminuria, Estimated-glomerular filtration rate, Cross-sectional study

## Abstract

**Aims:**

Examining the connection between the triglyceride-glucose (TyG) index and chronic kidney disease (CKD) was the aim of this investigation.

**Methods:**

Data from the National Health and Nutrition Examination Survey (NHANES) covering the years 1999–2020 were analyzed in this study. The TyG index was calculated as Ln (triglycerides (mg/dl) * fasting glucose (mg/dl)/2). The two criteria used to diagnose CKD were low estimated glomerular filtration rate (eGFR) (eGFR < 60 mL/min/1.73m^2^) or albuminuria (urine albumin-to-creatinine ratio (ACR) ≥ 30 mg/g). To look into the independent associations between TyG index levels with CKD, albuminuria, and low-eGFR, weighted multivariable logistic regression and generalized additive models were employed. To assess and contrast the diagnostic ability, receiver operating characteristic (ROC) curves were employed.

**Results:**

Out of 18,078 total participants recruited, 48.54% were male. 8.48 + 0.68 was the mean value of the TyG index. CKD, albuminuria, and low-eGFR were common, with respective prevalences of 17.06%, 11.26%, and 8.03%, respectively. The TyG index and CKD were observed to positively correlate (OR = 4.03; 95% CI 1.81, 8.96). In US adults between the ages of 41 and 60, a J-shaped connection was found between the two. Furthermore, a higher TyG index is associated with a higher prevalence of albuminuria (OR = 6.11; 95% CI 2.64, 14.14). Subgroup analyses and interaction tests revealed that different stratifications did not significantly affect the relationship between TyG index and CKD, albuminuria, and low-eGFR. Comparing the TyG index to other indicators [lipid accumulation product (LAP), Visceral adiposity index (VAI), and the triglyceride glucose–body mass index (TyG-BMI)], it may be more accurate and discriminative in predicting CKD and albuminuria.

**Conclusion:**

When predicting CKD and albuminuria, the TyG index may be a more useful marker when compared to other markers (LAP, VAI, and TyG-BMI index). In addition, in American adults aged 41–60, the TyG index shows a J-shaped relationship with CKD. As a result, when assessing the kidney health of US adults, we must pay close attention to the significance of the TyG index.

**Supplementary Information:**

The online version contains supplementary material available at 10.1007/s11255-024-04103-8.

## Introduction

Chronic kidney disease (CKD) is becoming more common, with the majority of cases being caused by diabetes and hypertension. It affects 15–20% of adults globally, raising the possibility of unfavorable outcomes [1–3]. Globally, CKD is the main source of catastrophic health expenditures, or medical costs that are more than 40% of household income [4]. By 2040, it is expected to rise to the fifth rank among the world’s major causes of death [5]. Thus, renal health is something we should take very seriously. According to studies, CKD is significantly influenced by insulin resistance (IR) [6, 7]. For clinical purposes, the hyperinsulinemic-normoglycemic clamp test, which is the gold standard for evaluating IR, is too time-consuming and expensive [8]. Therefore, alternative markers for IR have emerged.

Using fasting triglycerides and glucose, one may quickly and simply determine IR by calculating the triglyceride-glucose (TyG) index [9]. The TyG index can predict the occurrence of CKD, according to earlier studies. A cohort research involving 11,712 Japanese participants discovered a link between a higher TyG index and a greater prevalence of CKD [10]. TyG index and CKD prevalence were reported to be positively correlated in Chinese hypertension patients by Shi et al. [11]. TyG index and end-stage kidney disease (ESKD) were found to be positively correlated in an Austrian study [12]. Previous studies also explored the significant association between high levels of the TyG index and high levels of CKD and albuminuria in the US population [13, 14]. However, there are no studies that delve into the potential association between the TyG index and CKD in US adults while also assessing its predictive value for kidney disease.

As such, this study aims to investigate the relationship between the TyG index and CKD using data from the National Health and Nutrition Examination Survey (NHANES).

## Materials and methods

### Study design and population

**Fig. 1:**
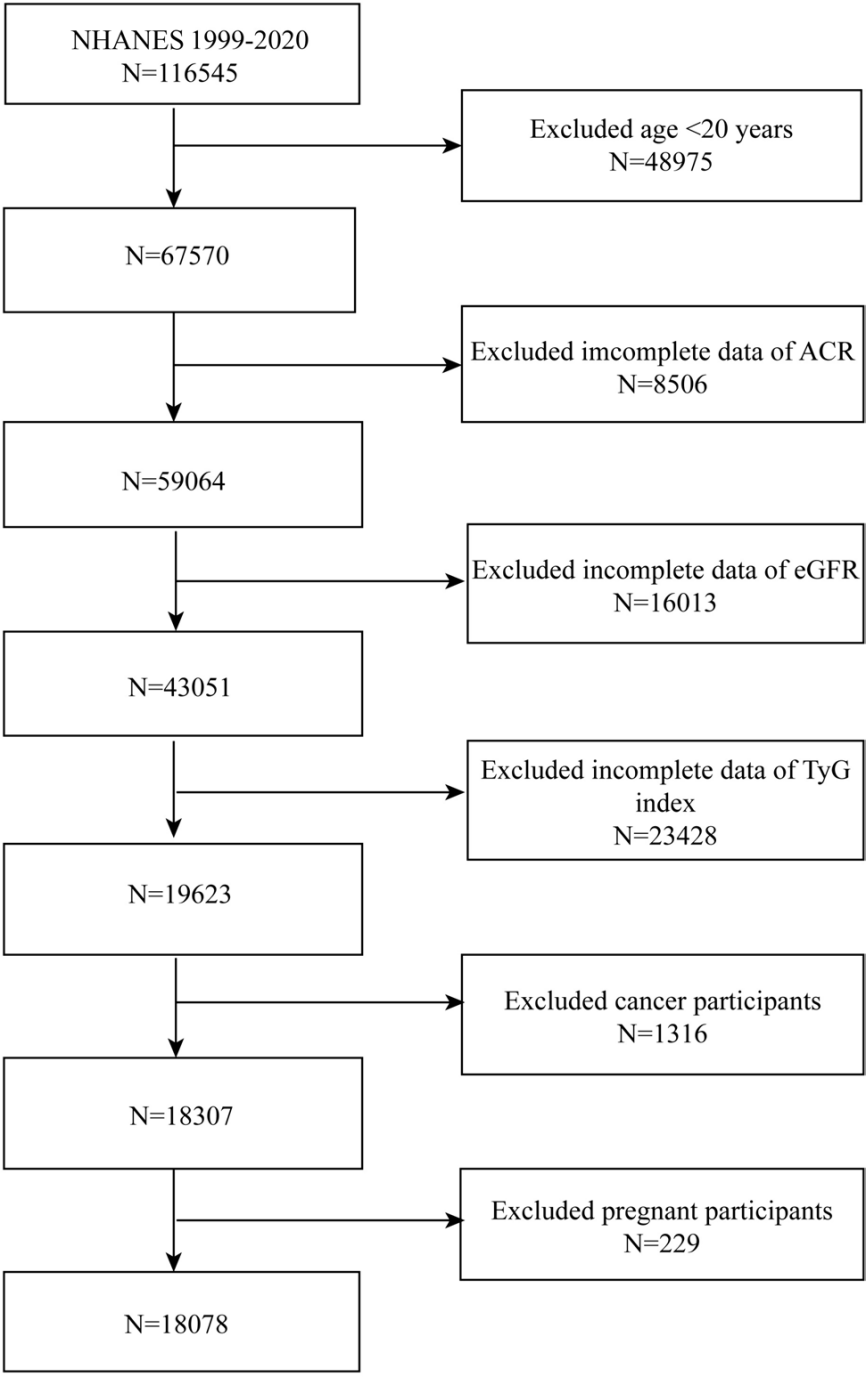
Flowchart of the sample selection from NHANES 1999–2020

NHANES is a research study that gathers health and nutrition information from US households through a population-based inquiry [15]. The NHANES 1999–2020 was the source of participants for our research. There were 18,078 eligible individuals who remained in the trial after patients who were < 20 years of age (*n* = 48,975), had cancer (*n* = 1316), were pregnant (*n* = 229), and lacked information regarding ACR (*n* = 8506), eGFR (*n* = 16,013), and TyG index (*n* = 23,428) were eliminated (Fig. 1). Both the NHANES survey protocols and the informed consent forms signed by each study participant were approved by the National Center for Health Statistics (NCHS) research ethics review committee.

### Definition of the TyG index and CKD

The TyG index, which was derived using Ln [triglycerides (mg/dl) * fasting glucose (mg/dl)/2], was considered as an exposure variable [16]. According to the NHANES protocol, enzymatic assays were used in an automatic biochemistry analyzer to measure triglycerides and fasting glucose. We also compare the predictive power of TyG index with other markers such as Visceral adiposity index (VAI)(male [WC/39.68 + (1.88 × BMI)] × (TG/1.03) × (1.31/HDL-C); female [WC/36.58 + 1.89 × (BMI)] × (TG/0.81) × (1.52/HDL-C)), lipid accumulation product (LAP)(male [WC − 65] × TG; female [WC − 58] × TG), and triglyceride glucose–body mass index (TyG-BMI) (TyG index × BMI) [17, 18].

To diagnose CKD, an albuminuric or eGFR of less than 60 mL/min/1.73 m^2^ is necessary [19]. In 2009, eGFR was determined using the Chronic Kidney Disease Epidemiology Collaboration (CKD-EPI) algorithm for standardized creatinine [20]. ACR ≥ 30 mg/g was used to characterize albuminuria. In our study, low-eGFR, albuminuria, and CKD were the outcome variables. During an individual interview, a standardized medical condition questionnaire was used to gather self-reported physician diagnoses, which led to the diagnosis of cardiovascular disease (CVD). The inquiry posed to the participants was, “Have you ever received a diagnosis of congestive heart failure, coronary heart disease, angina pectoris, myocardial infarction, or stroke from a doctor or other health expert?” Answering “yes” to any of the aforementioned questions was considered an indication of CVD.

### Selection of covariates

We adjusted for a number of demographic factors in our analysis, such as education level, race, age, and sex. We also included a number of laboratory and anthropometric covariates, including BMI, waist circumference (WC), smoking status, alcohol drinking (number of days of alcohol consumption in the past year), systolic blood pressure (SBP), diastolic blood pressure (DBP), aspartate aminotransferase (AST), alanine aminotransferase (ALT), serum uric acid, total cholesterol (TC), high-density lipoprotein cholesterol (HDL-C), low-density lipoprotein cholesterol (LDL-C) and serum total calcium.

Variations in health status, such as diabetes and hypertension, were also included as factors in our analysis. This study defines hypertension using three different criteria. “Ever told you you had hypertension” is a questionnaire item that serves as the basis for an assessment. In the second section, the mean diastolic or systolic blood pressure exceeding 130 or 80 mmHg is measured [21]. Using the item “taking hypertension prescription” program, participants with hypertension are identified in the third section. Three components were also included in the definition that was applied to diabetes. The first portion concerned self-reported diabetes, whereas the second concerned the use of insulin or other diabetes treatments. The last step was determining which patients had diabetes based on hemoglobin A1c (HbA1c) (%) > 6.5 and fasting glucose (mmol/l) ≥ 7.0.

### Statistical analysis

In compliance with the recommendations given by the US Centers for Disease Control and Prevention (CDC), every statistical analysis takes into account the intricate sample design of a multi-stage cluster survey [22]. The standard deviation and mean were displayed for continuous values, and percentages were used for categorical variables. For either continuous or categorical data, differences between groups of the TyG index (tertiles) were analyzed using weighted chi-square tests or *t* tests. A weighted multivariable regression model was used to investigate three different models that looked at the link between the TyG index and low-eGFR, albuminuria, and CKD. There was no covariate adjustment made in Model 1. Age, sex, and race adjustments were made to Model 2. Sex, age, race, BMI, WC, education level, smoking status, alcohol consumption, SBP, DBP, AST, ALT, serum uric acid, TC, LDL-C, HDL-C, serum total calcium, hypertension, and diabetes status were among the variables that were taken into consideration while adjusting Model 3. The non-linear problems were solved by smooth curve fitting and generalized additive models (GAM). Using subgroup analysis and a stratified multivariable logistic regression model stratified by sex, age, BMI, hypertension, and diabetes, the relationship between TyG index and CKD, albuminuria, and low-eGFR was also investigated. The predictive efficacy of the TyG index and other markers (LAP, VAI, and TyG-BMI index) was further evaluated using receiver operating characteristic (ROC) curves and area under the curve (AUC) values. Based on the data available, mode imputation was applied to missing values in categorical variables and median imputation to missing values in continuous variables. R 4.1.3 was used for all statistical analyses, along with the Empower software. One utilized a two-tailed *p* value of less than 0.05 to determine statistical significance.

## Results

### Participants characteristics at baseline

18,078 participants in total, 51.46 percent female and 48.54% male, with an average age of 48.93 ± 18.24 years for the analysis. 8.48 ± 0.68 was the average TyG index value. For prevalence of albuminuria it was 11.26%, for CKD it was 17.06%, and for low-eGFR it was 8.03%. Individuals in the upper tertile of the TyG index had greater rates of CKD, albuminuria, and low-eGFR prevalence (all *p* < 0.05). Age, BMI, WC, smoking status, hypertension, diabetes, SBP, DBP, TC, HDL-C, LDL-C, ACR, eGFR, albuminuria, low-eGFR, and CKD stages were all significantly different between tertiles (all *p* < 0.05)(Table 1). We also looked at the characteristics of the population which are the unavailability of data related to the TyG index (*n* = 23,428) (Supplementary Table S1).

**Table 1:** Baseline characteristics according to TyG index tertiles

TyG index	Overall	Tertile 1	Tertile 2	Tertile 3	*p *value
		(5.65–8.16)	(8.16–8.72)	(8.72–12.84)	
*N*	18,078	6023	6027	6028	
TyG index	8.48 ± 0.68	7.78 ± 0.29	8.44 ± 0.16	9.23 ± 0.47	< 0.001
Age, years					< 0.001
20–40	6808 (37.66%)	2147 (35.65%)	2314 (38.39%)	2347 (38.93%)	
41–60	5802 (32.09%)	1896 (31.48%)	1932 (32.06%)	1974 (32.75%)	
> 60	5468 (30.25%)	1980 (32.87%)	1781 (29.55%)	1707 (28.32%)	
Sex, *n* (%)					0.302
Male	8775 (48.54%)	2876 (47.75%)	2939 (48.76%)	2960 (49.10%)	
Female	9303 (51.46%)	3147 (52.25%)	3088 (51.24%)	3068 (50.90%)	
Race, *n* (%)					0.245
Mexican American	3065 (16.95%)	999 (16.59%)	1033 (17.14%)	1033 (17.14%)	
Other Hispanic	1558 (8.62%)	489 (8.12%)	524 (8.69%)	545 (9.04%)	
Non-Hispanic White	7817 (43.24%)	2635 (43.75%)	2631 (43.65%)	2551 (42.32%)	
Non-Hispanic Black	3845 (21.27%)	1306 (21.68%)	1274 (21.14%)	1265 (20.99%)	
Other Races	1793 (9.92%)	594 (9.86%)	565 (9.37%)	634 (10.52%)	
Education level, *n* (%)					0.948
Less than high school	4690 (25.95%)	1562 (25.93%)	1572 (26.09%)	1556 (25.82%)	
High school or GED	4126 (22.83%)	1365 (22.66%)	1383 (22.95%)	1378 (22.86%)	
Above high school	9216 (50.99%)	3085 (51.22%)	3055 (50.71%)	3076 (51.04%)	
Others	43 (0.24%)	11 (0.18%)	15 (0.25%)	17 (0.28%)	
Smoking status, *n* (%)					< 0.001
≥ 100 cigarettes lifetime	6291 (44.56%)	1329 (35.29%)	2128 (44.20%)	2834 (51.17%)	
< 100 cigarettes lifetime	7827 (55.44%)	2437 (64.71%)	2686 (55.80%)	2704 (48.83%)	
BMI, kg/m^2^					< 0.001
Normal weight	6939 (38.73%)	3516 (58.84%)	2270 (37.93%)	1153 (19.36%)	
Overweight	5433 (30.32%)	1426 (23.86%)	1925 (32.16%)	2082 (34.96%)	
Obese	5545 (30.95%)	1034 (17.30%)	1790 (29.91%)	2721 (45.69%)	
CKD stages, *n* (%)					< 0.001
1	1065 (34.52%)	362 (46.35%)	299 (32.71%)	404 (29.06%)	
2	569 (18.44%)	122 (15.62%)	162 (17.72%)	285 (20.50%)	
3	1339 (43.40%)	280 (35.85%)	421 (46.06%)	638 (45.90%)	
4	81 (2.63%)	12 (1.54%)	24 (2.63%)	45 (3.24%)	
5	31 (1.00%)	5 (0.64%)	8 (0.88%)	18 (1.29%)	
WC, cm	94.64 ± 17.27	85.76 ± 15.43	94.89 ± 16.23	103.34 ± 15.45	< 0.001
Alcohol drinking status, days	4.29 ± 10.03	4.77 ± 11.96	4.34 ± 12.37	3.80 ± 3.66	0.070
Hypertension, n (%)	9086 (50.26%)	2628 (43.63%)	2962 (49.15%)	3496 (58.00%)	< 0.001
Diabetes, *n* (%)	8399 (46.46%)	2677 (44.45%)	2469 (40.97%)	3253 (53.96%)	< 0.001
SBP, mmHg	123.40 ± 19.39	124.13 ± 19.95	123.36 ± 19.42	122.63 ± 18.68	0.001
DBP, mmHg	73.76 ± 10.70	73.10 ± 10.56	73.97 ± 10.98	74.28 ± 10.53	< 0.001
Serum uric acid, mg/dL	5.42 ± 1.55	5.49 ± 1.56	5.39 ± 1.58	5.40 ± 1.51	0.322
TC, mg/dL	183.35 ± 41.88	164.26 ± 33.51	184.60 ± 38.43	201.17 ± 44.47	< 0.001
HDL-C, mg/dL	54.27 ± 15.46	60.81 ± 15.43	55.06 ± 14.66	46.88 ± 12.87	< 0.001
LDL-C, mg/dL	106.29 ± 34.78	92.79 ± 27.95	110.33 ± 33.49	116.09 ± 38.00	< 0.001
AST, U/L	25.01 ± 16.30	24.97 ± 14.38	24.93 ± 16.18	25.12 ± 18.16	0.809
ALT, U/L	24.93 ± 24.14	24.61 ± 18.49	25.19 ± 32.51	24.99 ± 18.64	0.459
Serum total calcium, mg/dL	9.48 ± 0.41	9.50 ± 0.43	9.46 ± 0.41	9.48 ± 0.39	0.057
ACR, mg/g	38.28 ± 270.00	22.13 ± 152.41	26.50 ± 168.00	66.19 ± 407.48	< 0.001
eGFR, *m*L/min/1.73 m^2^	96.83 ± 26.74	99.94 ± 25.18	96.32 ± 26.31	94.22 ± 28.32	< 0.001
Albuminuria, n (%)	2036 (11.26%)	528 (8.77%)	581 (9.64%)	927 (15.38%)	< 0.001
Low-eGFR, *n* (%)	1451 (8.03%)	297 (4.93%)	453 (7.52%)	701 (11.63%)	< 0.001
CKD, *n* (%)	3085 (17.06%)	781 (12.97%)	914 (15.17%)	1390 (23.06%)	< 0.001

*TyG* triglyceride-glucose index, *GED* general educational development, *BMI* body mass index, *CKD* chronic kidney disease, *WC* waist circumference, *SBP* systolic blood pressure, *DBP* diastolic blood pressure, *TC* total cholesterol, *HDL-C* high-density lipoprotein-cholesterol, *LDL-C* low-density lipoprotein cholesterol, *AST* aspartate aminotransferase, *ALT* alanine aminotransferase, *ACR* urinary albumin-to-creatinine ratio, *eGFR* urinary albumin-to-creatinine ratio

### The association between the TyG index and CKD

Table 2 shows the correlation between CKD and the TyG index. In both crude and minimally adjusted models, our findings show a positive connection between the two. The positive correlation remains stable (OR = 4.03; 95% CI 1.81, 8.96) following full adjustment, suggesting that there is a 3.03-fold increase in the prevalence of CKD among the subjects for every unit rise in the TyG index. For a sensitivity analysis, the continuous variable was additionally converted to a categorical variable (tertiles) using the TyG index. The prevalence of CKD was more common in the higher tertiles than in the lower tertiles of the TyG index (*p* for trend < 0.05).

**Table 2:** Associations between TyG index and CKD, albuminuria, and low-eGFR

	Crude model (model 1)^3^		Adjusted model (model 2)^4^		Adjusted model (model 3)^5^	
	OR^1^ (95% CI^2^)	*p *value	OR (95% CI)	*p *value	OR (95% CI)	*p *value
CKD						
TyG index as continuous variable	1.65 (1.56, 1.75)	< 0.0001	1.72 (1.63, 1.82)	< 0.0001	4.03 (1.81, 8.96)	0.0006
Tertile 1	Reference		Reference		Reference	
Tertile 2	1.20 (1.08, 1.33)	0.0005	1.24 (1.11, 1.37)	< 0.0001	1.93 (1.03, 3.60)	0.0387
Tertile 3	2.01 (1.83, 2.21)	< 0.0001	2.14 (1.94, 2.36)	< 0.0001	3.18 (1.31, 7.74)	0.0108
*P* for trend	< 0.0001		< 0.0001		0.0102	
Albuminuria						
TyG index as continuous variable	1.67 (1.56, 1.78)	< 0.0001	1.68 (1.57, 1.79)	< 0.0001	6.11 (2.64, 14.14)	< 0.0001
Tertile 1	Reference		Reference		Reference	
Tertile 2	1.11 (0.98, 1.26)	0.0973	1.12 (0.99, 1.26)	0.0822	2.18 (1.11, 4.28)	0.0234
Tertile 3	1.89 (1.69, 2.12)	< 0.0001	1.91 (1.70, 2.14)	< 0.0001	4.51 (1.79, 11.38)	0.0014
*P* for trend	< 0.0001		< 0.0001		0.0014	
Low-eGFR						
TyG index as continuous variable	1.65 (1.53, 1.78)	< 0.0001	1.82 (1.69, 1.97)	< 0.0001	0.97 (0.31, 3.00)	0.9589
Tertile 1	Reference		Reference		Reference	
Tertile 2	1.57 (1.35, 1.82)	< 0.0001	1.68 (1.44, 1.96)	< 0.0001	1.05 (0.41, 2.64)	0.9254
Tertile 3	2.54 (2.20, 2.92)	< 0.0001	2.96 (2.56, 3.43)	< 0.0001	1.46 (0.39, 5.49)	0.5725
*P* for trend	< 0.0001		< 0.0001		0.5705	

In sensitivity analysis, the TyG index was converted from a continuous variable to a categorical variable (tertiles)

^1^OR: Odd ratio

^2^95% CI: 95% confidence interval

^3^Model 1: No covariates were adjusted

^4^Model 2: Adjusted for age, gender, and race

^5^Model 3: Adjusted for gender, age, race, BMI, WC, education level, smoking, alcohol drinking, SBP, DBP, AST, ALT, serum uric acid, TC, LDL-C, HDL-C, serum total calcium, hypertension, and diabetes status

**Fig. 2:**
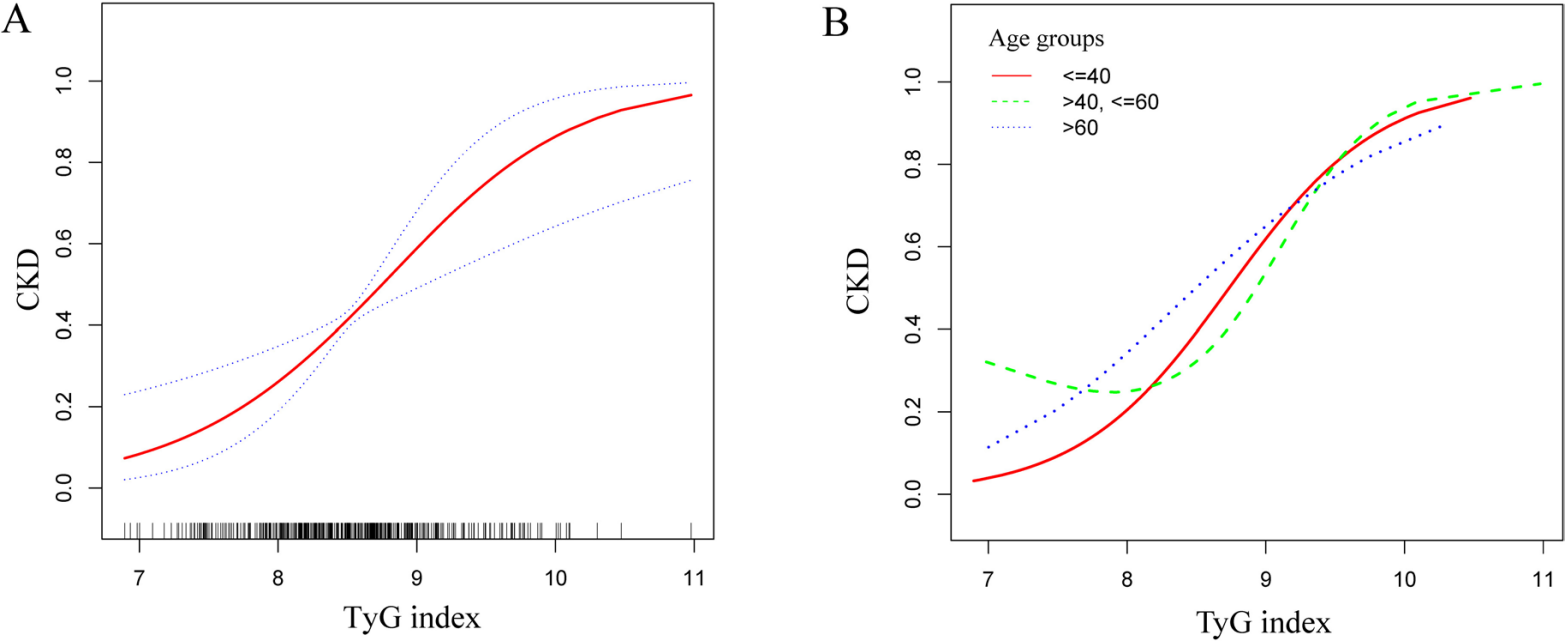
Smooth curve fitting for the TyG index and CKD. A non-linear relationship between the TyG index and CKD was detected by the generalized additive model in the group of people aged 41–60. **A** TyG index and CKD; **B** TyG index and CKD in different age groups

The TyG index and CKD did not indicate a non-linear connection, according to the smooth curve fitting (Fig. 2). Among those in the 41–60 age range, we discovered a non-linear correlation between the two. There was a definite breakpoint of 8.21. The TyG index and CKD did not seen to have a significant relationship with the breakpoint’s left side (OR = 0.68, 95% CI 0.37, 1.24). A positive correlation was seen between the two to the right of the breakpoint (OR = 6.04, 95% CI 1.60, 22.80) (Table 3).

**Table 3:** Threshold effect analysis of the TyG index on CKD using a two-piecewise linear regression model in different age groups

	< = 40	> 40, < = 60	> 60
Fitting by standard linear model			
OR^1^ (95% CI^2^)	2.99 (1.51, 5.92)	1.83 (0.84, 3.95)	3.08 (1.35, 7.00)
*p* value	0.0017	0.1256	0.0073
Fitting by two-piecewise linear model			
Breakpoint (K)	8.98	8.21	7.67
OR1 (< K)	2.63 (1.27, 5.45)	0.68 (0.37, 1.24)	0.83 (0.05, 13.89)
	0.0094	0.2106	0.897
OR2 (> K)	4.74 (1.49, 15.05)	6.04 (1.60, 22.80)	3.65 (1.48, 9.01)
	0.0084	0.0079	0.0049
OR2/OR1	1.80 (0.57, 5.74)	4.53 (1.23, 16.64)	4.40 (0.20, 95.37)
	0.3187	0.0227	0.3452
Logarithmic likelihood ratio test *p* value	0.313	0.019	0.354

Adjusted for gender, age, race, BMI, WC, education level, smoking, alcohol drinking, SBP, DBP, AST, ALT, serum uric acid, TC, LDL-C, HDL-C, serum total calcium, hypertension, and diabetes status

^1^OR: Odd ratio

^2^95% CI: 95% confidence interval

### The association between the TyG index and albuminuria

Additionally, a higher prevalence of albuminuria was observed to be correlated with a higher TyG index (OR = 6.11; 95% CI 2.64, 14.14). There was still a statistically significant link even after switching the TyG index to tertiles. The higher tertiles had a higher prevalence of albuminuria than the lower tertiles of the TyG index, respectively (*p* for trend < 0.05) (Table 2).

**Fig. 3:**
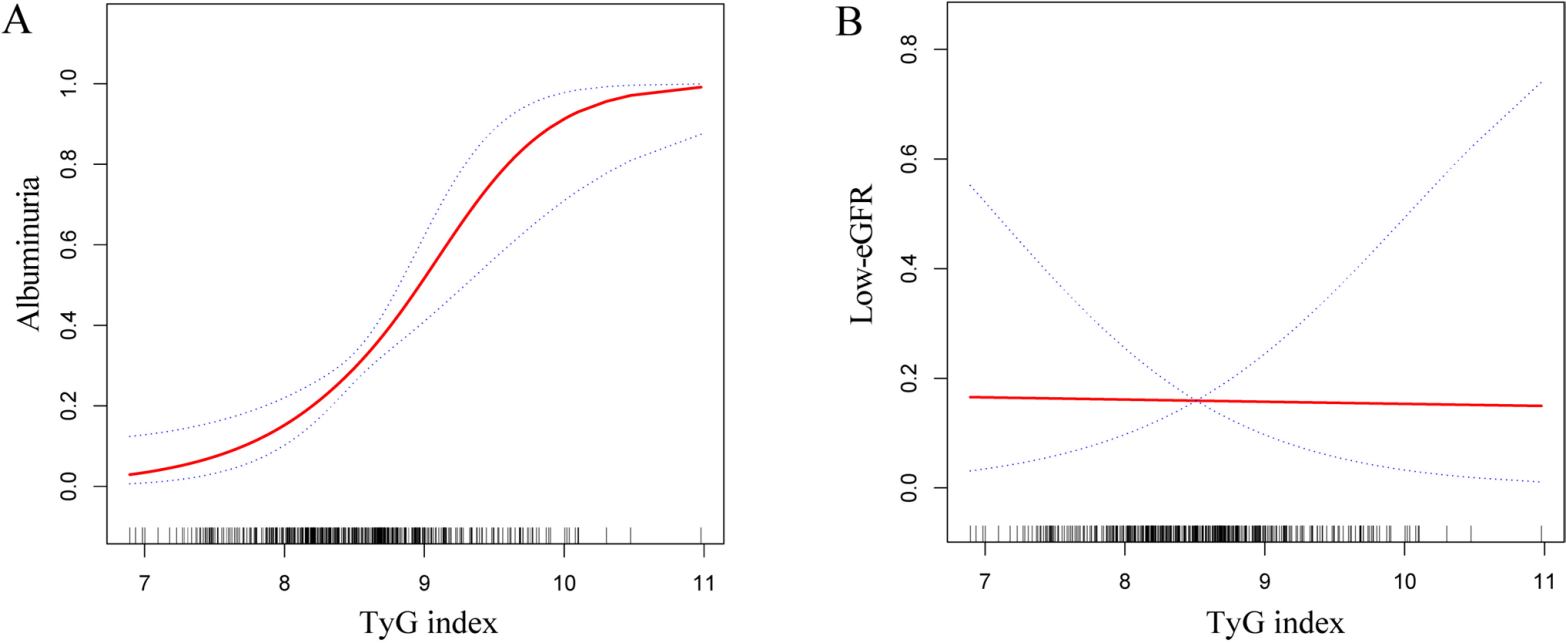
Smooth curve fitting for the TyG index and albuminuria and low-eGFR. A non-linear relationship between the TyG index and albuminuria was detected by the generalized additive model. **A** TyG index and albuminuria; **B** TyG index and low-eGFR

The TyG index and albuminuria were revealed to have a non-linear association based on GAM and smooth curve fitting (Fig. 3). Our calculations resulted in a breakpoint of 8.72. The TyG index and albuminuria were positively correlated on either side of the breakpoint (left side: OR = 4.19, 95% CI 1.68, 10.47; right side: OR = 17.16, 95% CI 4.24, 69.46) (Table 4).

**Table 4:** Threshold effect analysis of the TyG index on albuminuria using a two-piecewise linear regression model before and after adjustment of covariates

Albuminuria	Before adjustment^3^	After adjustment^4^
Fitting by standard linear model		
OR^1^ (95%CI^2^)	1.67 (1.56, 1.78)	6.11 (2.64, 14.14)
*p* value	< 0.0001	< 0.0001
Fitting by two-piecewise linear model		
Breakpoint (K)	8.64	8.72
OR1 (< K)	1.08 (0.95, 1.23)	4.19 (1.68, 10.47)
	0.2588	0.0022
OR2 (> K)	2.25 (2.03, 2.49)	17.16 (4.24, 69.46)
	< 0.0001	< 0.0001
OR2/OR1	2.08 (1.70, 2.55)	4.10 (0.98, 17.03)
	< 0.0001	0.0525
Logarithmic likelihood ratio test *p* value	< 0.001	0.047

^1^OR: Odd ratio

^2^95% CI: 95% confidence interval

^3^Before adjustment: No adjustment

^4^After adjustment: Adjusted for gender, age, race, BMI, WC, education level, smoking, alcohol drinking, SBP, DBP, AST, ALT, serum uric acid, TC, LDL-C, HDL-C, serum total calcium, hypertension, and diabetes status

### The association between the TyG index and low-eGFR

The TyG index and low-eGFR were found to be significantly positively correlated in both the crude model (Model 1: OR = 1.65, 95% CI 1.53, 1.78) and the minimally adjusted model (Model 2: OR = 1.82, 95% CI 1.69, 1.97). The positive connection did not, however, achieve statistical significance after full adjustment (OR = 0.97, 95% CI 0.31, 3.00) (Table 2).

The TyG index and low-eGFR did not show any non-linear connection, according to smooth curve fitting (Fig. 3).

### Subgroup analysis

**Fig. 4:**
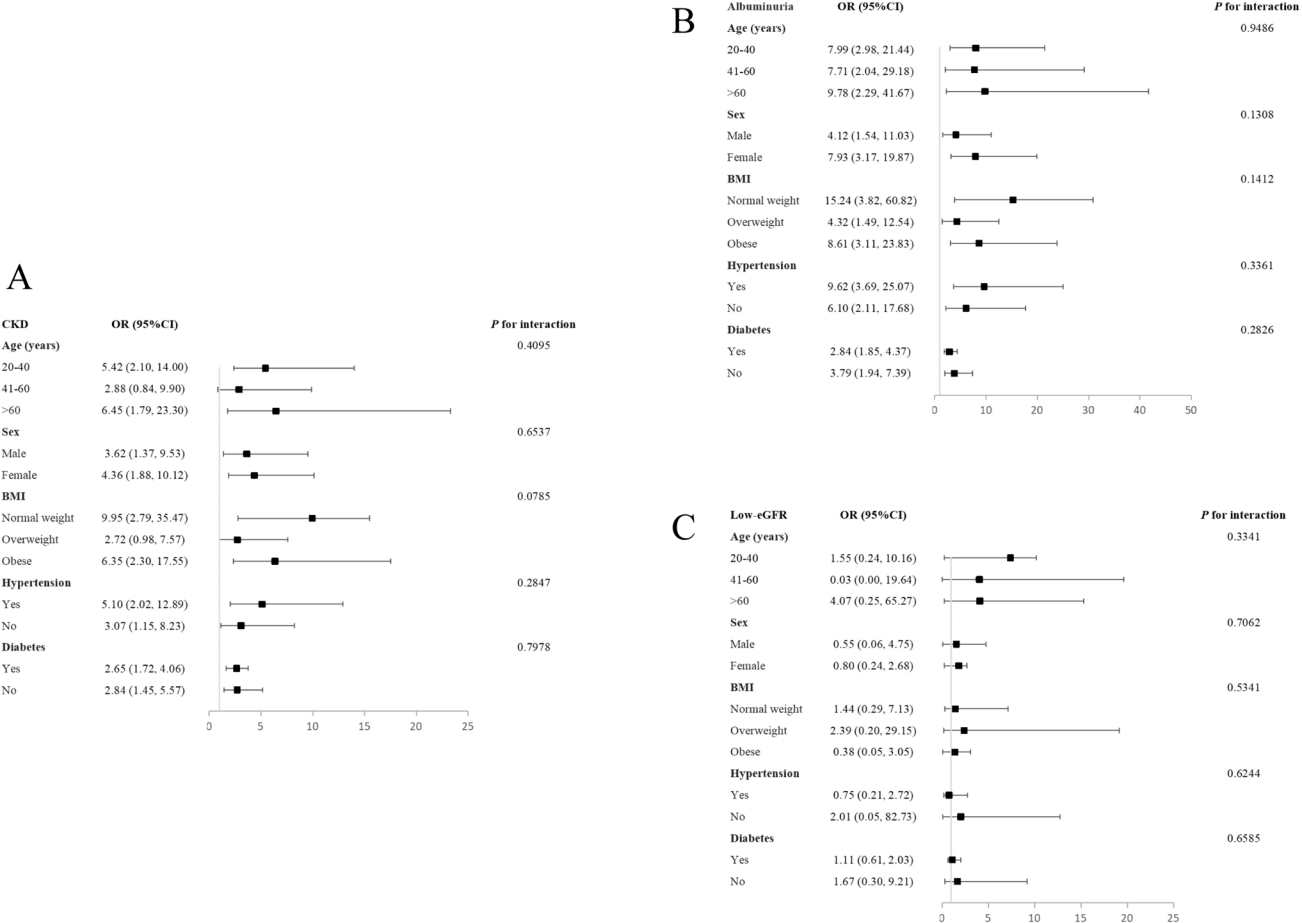
Subgroup analysis for the associations between the TyG index and CKD, albuminuria, and low-eGFR. **A** TyG index and CKD; **B** TyG index and albuminuria; **C** TyG index and low-eGFR

CKD, albuminuria, and low-eGFR are not consistently correlated with the TyG index, according to our findings. TyG index and CKD were found to have significant relationships in each subgroup broken down by sex, diabetes, and hypertension (all *p* < 0.05). In the overweight and 41–60 age group, the TyG index did, however, demonstrate a positive but non-significant relationship with CKD. The interaction tests demonstrated that there was no significant influence of age, sex, BMI, hypertension, or diabetes on the connection between the TyG index with CKD, albuminuria, and low-eGFR (all *p* for interaction > 0.05) (Fig. 4).

### ROC analysis

**Fig. 5:**
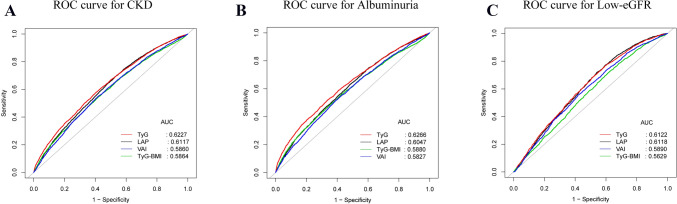
ROC curves and the AUC values of the four markers (TyG index, LAP, VAI, and TyG-BMI index) in diagnosing CKD, albuminuria and low-eGFR. **A** Four inflammatory markers were assessed to identify CKD. **B** Four inflammatory markers were assessed to identify albuminuria. **C** Four inflammatory markers were assessed to identify low-eGFR

For CKD, albuminuria, and low-eGFR, we calculated the AUC values to assess the prediction accuracy of the TyG index with other markers (LAP, VAI, and TyG-BMI index) (Fig. 5). Comparing the TyG index to the other indicators, our results show that it had higher AUC values. Furthermore, Table 4 demonstrates that the TyG index and other indicators had statistically significant differences in AUC values in the prediction of CKD and albuminuria (all *p* < 0.05) (Table 5). These results demonstrate that when compared to other indicators (LAP, VAI, and TyG-BMI index), the TyG index may have a greater discriminative capacity and accuracy in predicting CKD and albuminuria.

**Table 5:** Comparison of AUC values between TyG index and other markers for predicting CKD, albuminuria, and low-eGFR

Test	AUC^1^	95% CI^2^ low	95% CI upp	Best threshold	Specificity	Sensitivity	*p* for different in AUC
CKD							
TyG index	0.6227	0.6129	0.6324	8.6894	0.5937	0.5804	Reference
LAP	0.6117	0.6017	0.6216	37.8734	0.4716	0.6923	< 0.0001
VAI	0.5864	0.5762	0.5966	1.6548	0.5841	0.5475	< 0.0001
TyG-BMI index	0.5860	0.5757	0.5962	251.8171	0.5838	0.5424	< 0.0001
Albuminuria							
TyG index	0.6122	0.5988	0.6256	8.4094	0.4048	0.7679	Reference
LAP	0.6118	0.5981	0.6255	38.0713	0.4611	0.7177	< 0.0001
VAI	0.5890	0.5745	0.6034	1.6648	0.5813	0.5558	< 0.0001
TyG-BMI index	0.5629	0.5487	0.5770	222.2815	0.3761	0.7270	< 0.0001
Low-eGFR							
TyG index	0.6266	0.6151	0.6381	8.8205	0.6607	0.5212	Reference
LAP	0.6047	0.5931	0.6163	42.8790	0.5185	0.6300	0.9644
VAI	0.5827	0.5708	0.5945	1.7680	0.6115	0.5147	< 0.0001
TyG-BMI index	0.5880	0.5760	0.6001	253.1372	0.5867	0.5464	< 0.0001

^1^AUC: area under the curve

^2^95% CI: 95% confidence interval

### The association between the TyG index and CVD

We looked more closely at the connection between CVD and the TyG index. The two were found to positively correlate (Supplementary Table S2). We used smooth curve fitting and GAM to find the nonlinear relationship between the two (Supplementary Fig. S1). Supplementary Table S3 shows that 9.52 was its breakpoint. The interaction test results showed that age, sex, BMI, hypertension, or diabetes did not significantly affect the link between the two (Supplementary Fig. S2). ROC analysis revealed that for CVD prediction, the TyG index performed better in terms of AUC values than the LAP, VAI, and TyG-BMI index (Supplementary Fig. S3, Table S4).

## Discussion

The prevalences of CKD and albuminuria was positively correlated with the TyG index level in this cross-sectional investigation of 18,078 adults. Additionally, we discovered a J-shaped connection between the TyG index and CKD in the 41–60 age range (*K* = 8.21). If, that is, the TyG index was greater than 8.21, the prevalence of CKD in the 41–60 age group of American participants increased significantly. There was no discernible impact of population differences on the relationship between the TyG index and low-eGFR, albuminuria, and CKD, according to subgroup analysis and interaction testing. Additionally, in comparison to other markers (LAP, VAI, and TyG-BMI index), the TyG index may have greater discriminative power and accuracy in predicting CKD and albuminuria.

In several populations and regions, the link between the TyG index and CKD has been researched [23–25]. One study found a connection between a rise in the TyG index and deteriorating kidney function in senior Chinese persons [26]. The TyG index and ESKD (end-stage renal disease) were found to be significantly correlated in cohort research from Austria [12]. In a cohort analysis of 11,712 patients in Japan, the TyG index was found to have a positive relationship with CKD [10]. In China, the TyG index and CKD of hypertension patients were positively associated [11]. A higher TyG index was linked to a higher prevalence of CKD and albuminuria, according to US studies [13, 14]. Our study has a number of advantages over earlier studies as well. Firstly, in the US population of people aged 41–60, which has received less attention in other studies on kidney health in middle-aged adults, our study discovered for the first time a J-shaped link between the TyG index and CKD. Secondly, whereas earlier research on the connection between low-eGFR and the TyG index has produced contentious findings, our analysis of data on US adults revealed no significant connection between the two [10, 13, 26, 27]. Thirdly, we explored territory by conducting ROC analyses to assess the predictive abilities of the TyG index, LAP, VAI, and TyG-BMI index for CKD, albuminuria, and low-eGFR in US adults. This is a fundamental difference from previous studies. Additionally, our study delved into the TyG index’s predictive significance for cardiovascular disease (CVD) prevalence in US adults. This aspect, previously explored primarily for its prognostic implications, adds another layer to our understanding [28, 29].

Our study, in addition to showing a positive correlation between TyG index levels with CKD, demonstrated a J-shaped link between the two in the group of people aged 41–60 (*K* = 8.21). The twos had a negative correlation, although it was not statistically significant, on the left side of the breakpoint. Nonetheless, every unit increase in the TyG index was linked to a 5.04-fold rise in the prevalence of CKD on the right side of the breakpoint. Therefore, Americans aged 41–60 with a TyG index > 8.21 should pay close attention to their kidney health. TyG index and CKD have been established in earlier research to have a nonlinear association in patients with impaired glucose metabolism and hypertension [30]. A non-linear association between the TyG index and the prevalence of diabetic nephropathy was also discovered by Shang et al. [31]. However, we need more prospective studies to confirm our findings.

According to our research, the TyG index and albuminuria are positively and nonlinearly correlated in US people. The link was seen on both sides of a breakpoint (*K* = 8.72), with a high positive correlation on the right side and a much weaker positive correlation on the left. That is, the prevalence of albuminuria considerably rises when the TyG index is higher than 8.72. Nonlinear relationships between the TyG index and other diseases have been discovered in prior research. Jiang et al. found a nonlinear correlation between the TyG index and the prevalence of kidney stones [32]. Adult US deaths from cardiovascular and all-cause were shown to have a nonlinear relationship with the TyG index by Liu et al. [33]. The relationship between low-eGFR and the TyG index in previous studies remains controversial [10, 13, 26, 27]. We could not find any relationship between low-eGFR and the TyG index. We believe that different results can be obtained from different demographic characteristics, including population, race, area, sample size, and eGFR calculation method.

IR has been demonstrated to have a significant correlation with CKD [6, 7]. However, the gold standard test for diagnosing IR can be difficult to perform in clinical settings [8]. TyG index, as a simple and feasible IR indicator, has been shown to be significantly better than other traditional IR indicators. Research indicates that the TyG index outperforms the VAI and lipid accumulation product index (LAPI) in predicting CKD occurrence [34]. In our study, we also examined the TyG index’s superiority. ROC analysis showed that the TyG index was a more reliable indicator of CKD and albuminuria than other indicators (LAP, VAI, and TyG-BMI index). To sum up, the TyG index has a lot of potential for clinical use in CKD prediction and might be a more straightforward and accurate IR indicator.

CKD was more likely to occur in female participants, according to our subgroup analysis (Males: OR = 3.62, 95% CI 1.37, 9.53; Females: OR = 4.36, 95% CI 1.88, 10.12). This result has been supported by earlier research [10, 34, 35]. However, the fundamental cause of the sex gap is yet unknown, necessitating additional research. Importantly, the independent associations between the TyG index and CKD, albuminuria, or low eGFR persisted across age, sex, BMI, hypertension, and diabetes. These associations may be applicable to diverse populations, reinforcing the adverse impact of the TyG index on renal function.

Inflammation and oxidative stress brought on by IR may be the basis for the relationship between the TyG index and CKD. IR inhibits the insulin signaling pathway, causes a rise in monocyte chemoattractant protein-1 (MCP-1) synthesis, and encourages inflammation in adipose tissue. Tumor necrosis factor (TNF) -alpha and interleukin-6 (IL-6) are two pro-inflammatory cytokines that are produced when macrophages are activated by inflammatory stimuli in adipose tissue [36, 37]. Endothelial dysfunction is related to CKD and is facilitated by TNF-alpha and IL-6 [10, 38]. Additionally, oxidative stress and IR are related [38]. The activation of nuclear factor erythroid-2-related factor-2 (Nrf-2), which defends renal tissue, might be compromised by oxidative stress and inflammation [39]. The precise mechanisms underlying this relationship, however, need more research.

CKD increases the risk of various adverse outcomes, especially CVD [40]. As a result, we looked into the possibility of a connection between CVD and the TyG index. According to our findings, for every unit rise in the TyG index, the prevalence of CVD increased by 61%. Similar findings were noted in earlier research [28, 41, 42]. Prior research also revealed that the TyG index outperformed VAI as a predictor of CVD risk [43]. In addition, our research revealed that, when compared to the LAP, VAI, and TyG-BMI index, the TyG index had the greatest AUC value for predicting CVD. Therefore, we need to pay equal attention to the importance of the TyG index for renal and cardiovascular health in US adults.

The advantages of our research are numerous. Initially, the NHANES data is a nationwide population-based survey. The second reason is that our study is more reliable and representative because of its large sample size and adjustment for confounding factors. However, there are several shortcomings in our investigation. The cross-sectional design, for example, made it unable to show a causal relationship between the TyG index and CKD. Second, we are unable to completely exclude the influence of additional potential confounding variables, even after adjusting for a number of significant confounders. Third, because the US population survey NHANES is cross-sectional in nature, extrapolating our findings to other ethnic groups or the larger population may prove difficult.

## Conclusion

When predicting CKD and albuminuria, the TyG index may be a more useful marker when compared to other markers (LAP, VAI, and TyG-BMI index). In addition, in American adults aged 41–60, the TyG index shows a J-shaped relationship with CKD. As a result, when assessing the kidney health of US adults, we must pay close attention to the significance of the TyG index.

## Supplementary Information

Below is the link to the electronic supplementary material.

Supplementary Fig. S1 Smooth curve fitting for the TyG index and CVD (TIF 8093 KB)

Supplementary Fig. S2 Subgroup analysis for the associations between the TyG index and CVD (TIF 13083 KB)

Supplementary Fig S3 ROC curves and the AUC values of the five markers (LAP, VAI, and TyG-BMI index) in diagnosing CVD. (TIF 10040 KB)

Supplementary file4 (DOCX 18 KB)

Supplementary file5 (DOCX 14 KB)

Supplementary file6 (DOCX 13 KB)

Supplementary file7 (DOCX 14 KB)

## Data Availability

Publicly available datasets were analyzed in this study. This data can be found here: https://www.cdc.gov/nchs/nhanes/.
