# Supplementary figures and images for: Association between triglyceride-glucose index and chronic kidney disease: results from NHANES 1999–2020
Source: Int Urol Nephrol. 2024 Jun 10;56(11):3605–16. doi: 10.1007/s11255-024-04103-8 (PMC11464617; doi:10.1007/s11255-024-04103-8)

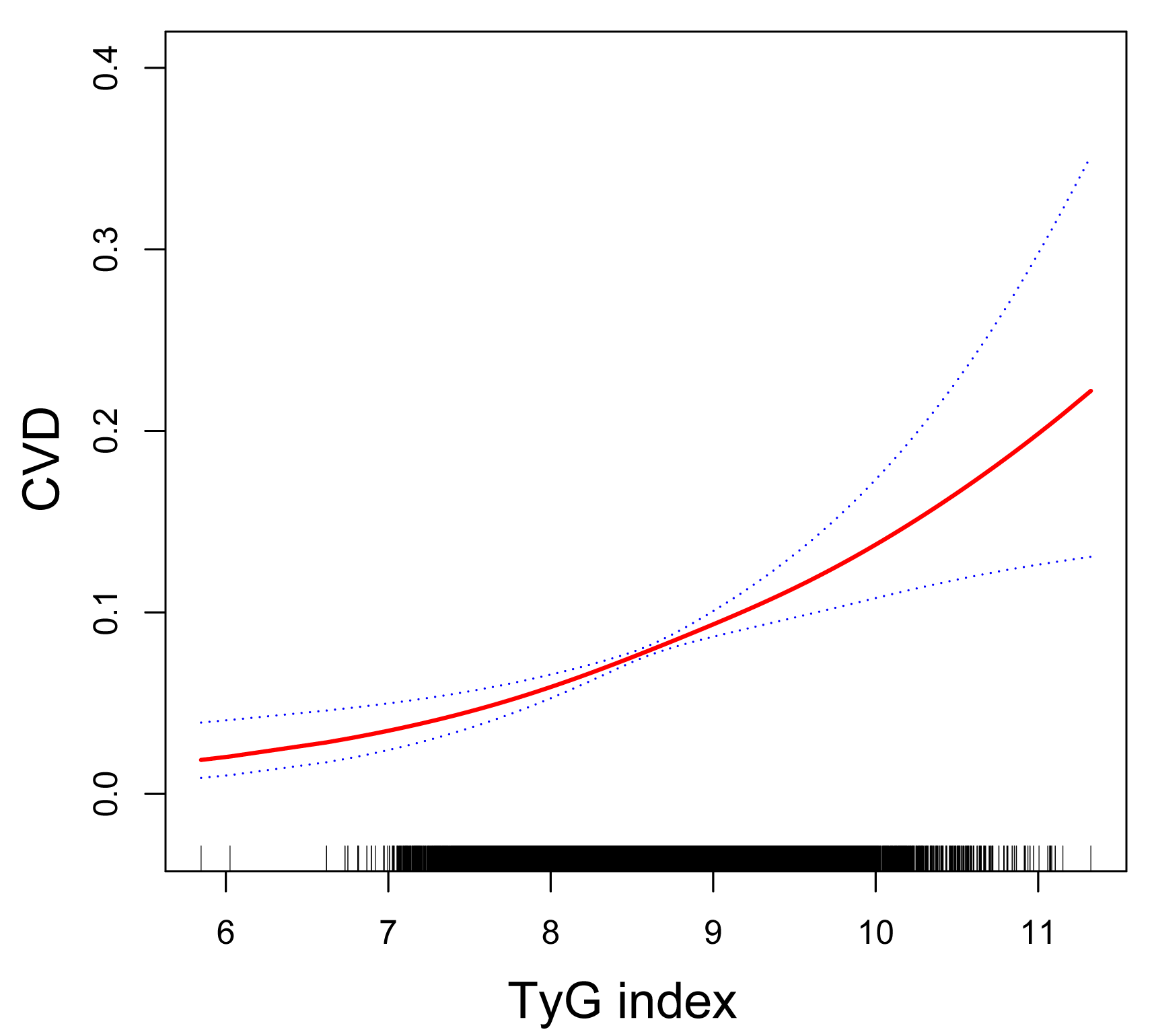

Supplement: Supplementary file 1 — Supplementary Fig. S1 Smooth curve fitting for the TyG index and CVD (TIF 8093 KB) [file 11255_2024_4103_MOESM1_ESM.tif]

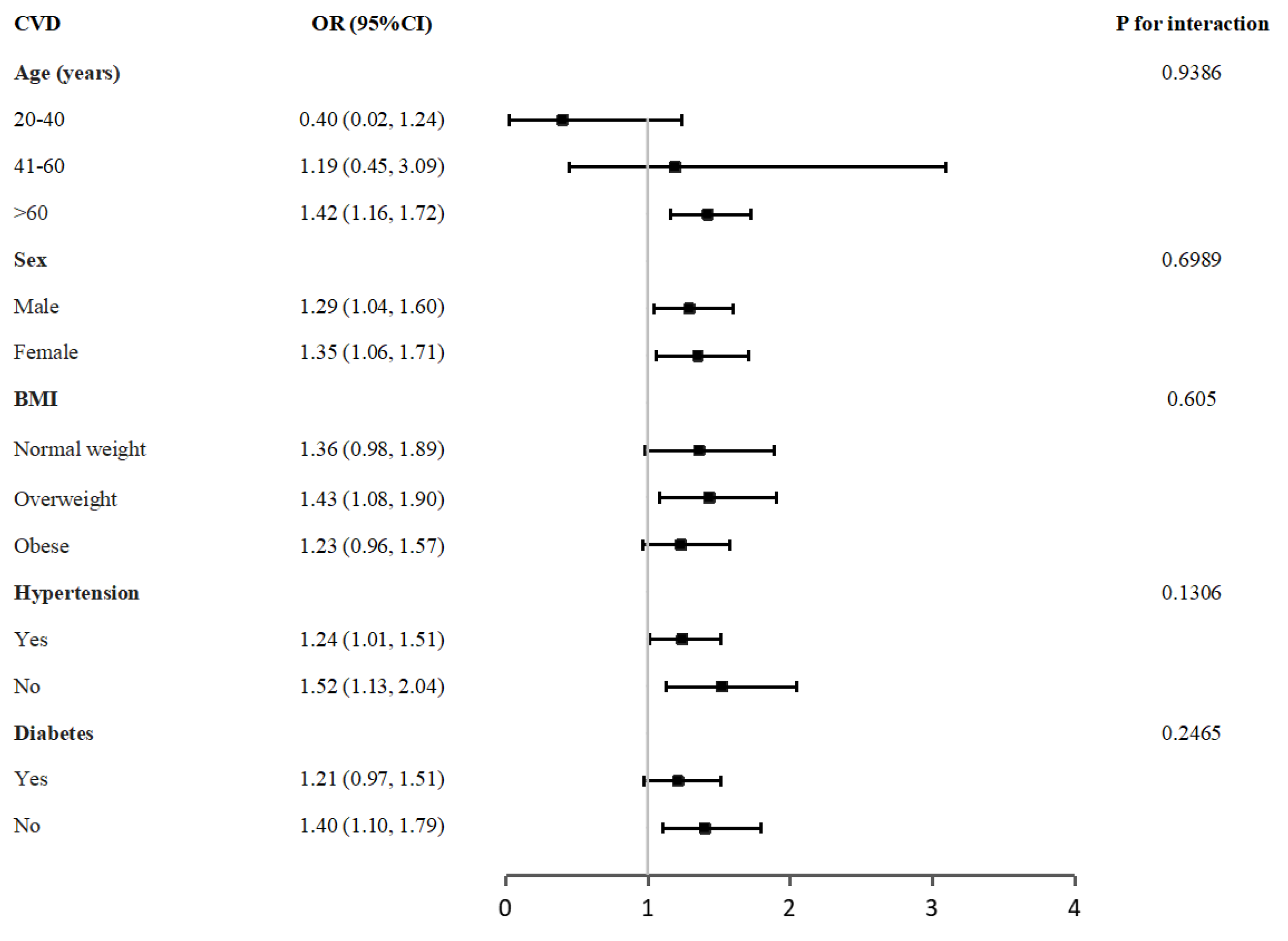

Supplement: Supplementary file 2 — Supplementary Fig. S2 Subgroup analysis for the associations between the TyG index and CVD (TIF 13083 KB) [file 11255_2024_4103_MOESM2_ESM.tif]

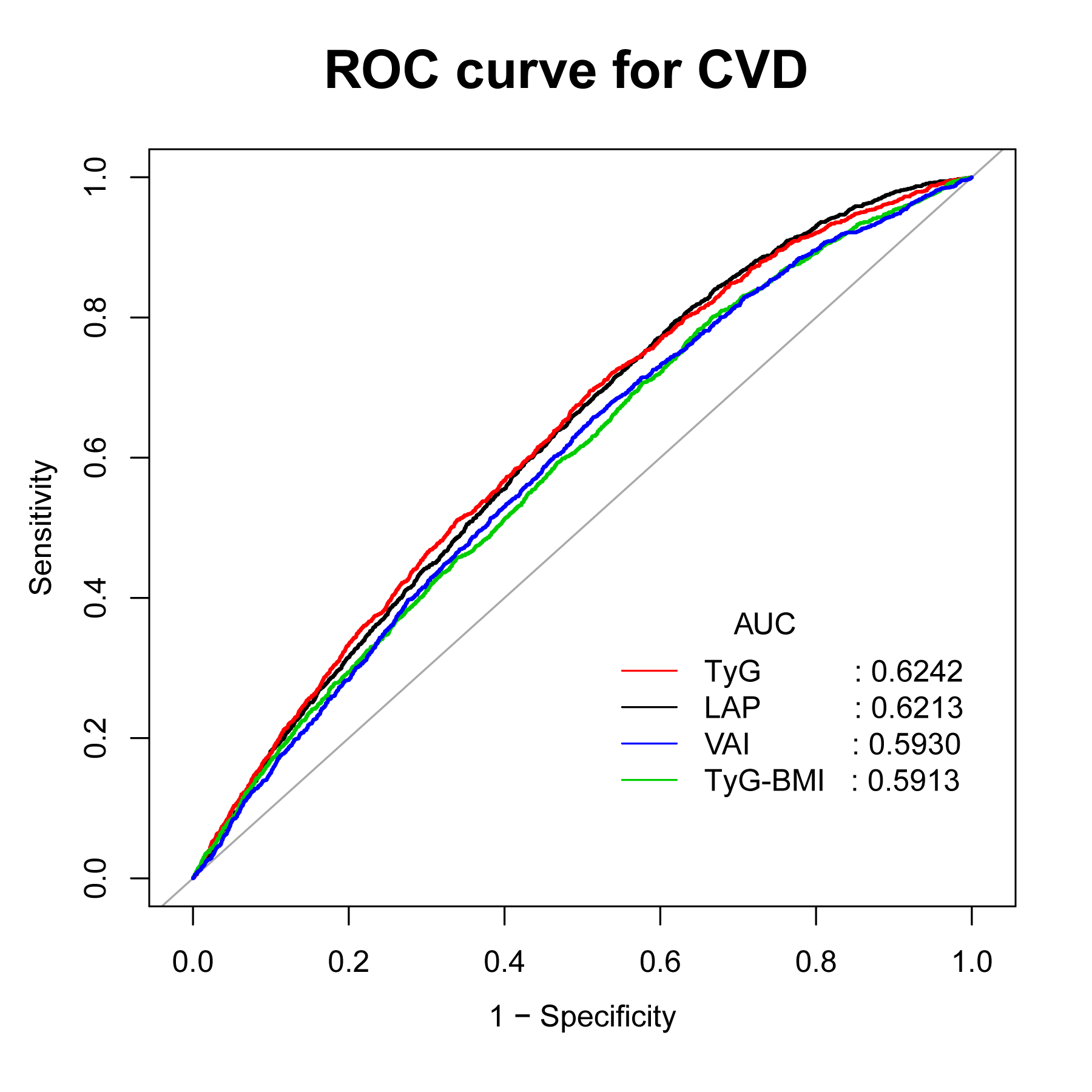

Supplement: Supplementary file 3 — Supplementary Fig S3 ROC curves and the AUC values of the five markers (LAP, VAI, and TyG-BMI index) in diagnosing CVD. (TIF 10040 KB) [file 11255_2024_4103_MOESM3_ESM.tif]
