# Supplementary material for: Association between triglyceride-glucose index and chronic kidney disease: results from NHANES 1999–2020
Source: Int Urol Nephrol. 2024 Jun 10;56(11):3605–16. doi: 10.1007/s11255-024-04103-8 (PMC11464617; doi:10.1007/s11255-024-04103-8)
Supplement: Supplementary file 4 — Supplementary file4 (DOCX 18 KB) [file 11255_2024_4103_MOESM4_ESM.docx]

**Supplementary Table S1 |** Baseline characteristics of the population which are the unavailability of data related to the TyG index.

| TyG index | Overall | CKD | Non-CKD | *P-*value |
| --- | --- | --- | --- | --- |
|  |  | (5.65–8.16) | (8.16–8.72) |  |
| N | 18078 | 3993 (17.04%) | 19435 (82.96%) |  |
| Age , years |  |  |  | <0.001 |
| 20-40 | 8908 (38.02%) | 1161 (29.08%) | 7747 (39.86%) |  |
| 41-60 | 7624 (32.54%) | 1249 (31.28%) | 6375 (32.80%) |  |
| > 60 | 6896 (29.43%) | 1583 (39.64%) | 5313 (27.34%) |  |
| Sex , n (%) |  |  |  | <0.001 |
| Male | 11261 (48.07%) | 1548 (38.77%) | 9713 (49.98%) |  |
| Female | 12167 (51.93%) | 2445 (61.23%) | 9722 (50.02%) |  |
| Race , n (%) |  |  |  | <0.001 |
| Mexican American | 4056 (17.31%) | 652 (16.33%) | 3404 (17.51%) |  |
| Other Hispanic | 2007 (8.57%) | 339 (8.49%) | 1668 (8.58%) |  |
| Non-Hispanic White | 10120 (43.20%) | 1861 (46.61%) | 8259 (42.50%) |  |
| Non-Hispanic Black | 4987 (21.29%) | 778 (19.48%) | 4209 (21.66%) |  |
| Other Races | 2258 (9.64%) | 363 (9.09%) | 1895 (9.75%) |  |
| Education level , n (%) |  |  |  | 0.008 |
| Less than high school | 6183 (26.39%) | 1074 (26.90%) | 5109 (26.29%) |  |
| High school or GED | 5421 (23.14%) | 987 (24.72%) | 4434 (22.82%) |  |
| Above high school | 11777 (50.27%) | 1921 (48.11%) | 9856 (50.72%) |  |
| Others | 45 (0.19%) | 11 (0.28%) | 34 (0.17%) |  |
| Smoking status, n (%) |  |  |  | <0.001 |
| ≥100 cigarettes lifetime | 7625 (44.12%) | 1586 (51.06%) | 6039 (42.59%) |  |
| < 100 cigarettes lifetime | 9659 (55.88%) | 1520 (48.94%) | 8139 (57.41%) |  |
| BMI , kg/m2 |  |  |  | <0.001 |
| Normal weight | 9402 (40.44%) | 1491 (37.90%) | 7911 (40.96%) |  |
| Overweight | 6916 (29.75%) | 1158 (29.44%) | 5758 (29.81%) |  |
| Obese | 6932 (29.82%) | 1285 (32.66%) | 5647 (29.23%) |  |
| WC, cm | 93.55 ± 17.08 | 96.10 ± 18.43 | 93.04 ± 16.75 | <0.001 |
| Alcohol drinking status, days | 4.07 ± 4.23 | 3.64 ± 4.67 | 4.16 ± 4.12 | 0.004 |
| Hypertension , n (%) | 11145 (47.57%) | 2476 (62.01%) | 8669 (44.61%) | < 0.001 |
| Diabetes, n (%) | 8399 (46.46%) | 2677 (44.45%) | 2469 (40.97%) | < 0.001 |
| SBP, mmHg | 123.57 ± 19.15 | 125.84 ± 20.71 | 123.11 ± 18.78 | <0.001 |
| DBP, mmHg | 73.81 ± 10.86 | 73.26 ± 11.64 | 73.92 ± 10.69 | 0.002 |
| Serum uric acid , mg/dL | 5.40 ± 1.55 | 5.32 ± 1.46 | 5.46 ± 1.61 | 0.005 |
| TC, mg/dL | 184.02 ± 41.57 | 181.20 ± 42.15 | 184.60 ± 41.43 | <0.001 |
| HDL-C, mg/dL | 52.45 ± 15.43 | 50.64 ± 15.34 | 52.86 ± 15.42 | <0.001 |
| LDL-C, mg/dL | 112.44 ± 35.12 | 115.37 ± 36.92 | 112.09 ± 34.89 | 0.208 |
| AST，U/L | 24.90 ± 20.16 | 24.72 ± 13.21 | 24.94 ± 21.31 | 0.570 |
| ALT，U/L | 24.97 ± 25.68 | 24.15 ± 17.88 | 25.14 ± 27.00 | 0.038 |
| Serum total calcium, mg/dL | 9.47 ± 0.42 | 9.47 ± 0.41 | 9.47 ± 0.43 | 0.710 |
| ACR , mg/g | 35.91 ± 278.73 | 171.15 ± 658.57 | 8.13 ± 5.68 | <0.001 |
| eGFR , mL/min/1.73 m2 | 99.58 ± 30.27 | 79.26 ± 36.00 | 103.76 ± 27.12 | <0.001 |

TyG, triglyceride-glucose index; GED, general educational development; BMI, body mass index; CKD, chronic kidney disease; WC, waist circumference;SBP, systolic blood pressure; DBP, diastolic blood pressure; TC, total cholesterol; HDL-C, high density lipoprotein-cholesterol; LDL-C, low-density lipoprotein cholesterol; AST, aspartate aminotransferase; ALT, alanine aminotransferase; ACR, urinary albumin-to-creatinine ratio; eGFR, urinary albumin-to-creatinine ratio.
