# Supplementary material for: Association between triglyceride-glucose index and chronic kidney disease: results from NHANES 1999–2020
Source: Int Urol Nephrol. 2024 Jun 10;56(11):3605–16. doi: 10.1007/s11255-024-04103-8 (PMC11464617; doi:10.1007/s11255-024-04103-8)
Supplement: Supplementary file 5 — Supplementary file5 (DOCX 14 KB) [file 11255_2024_4103_MOESM5_ESM.docx]

**Supplementary Table S2 |** Association between TyG index and CVD.

|  | Crude model (Model 1)3 | | Adjusted model(Model 2)4 | | [Adjusted model(Model 3)^5^](#t2fna) | |
| --- | --- | --- | --- | --- | --- | --- |
|  | OR1 (95% CI2) | *p-*value | OR (95% CI) | *p-*value | OR (95% CI) | *p-*value |
| **CVD** |  |  |  |  |  |  |
| TyG index as continuous variable | 1.73 (1.62, 1.85) | <0.0001 | 1.52 (1.41, 1.64) | <0.0001 | 1.31 (1.07, 1.59) | 0.0074 |
| Tertile 1 | Reference |  | Reference |  | Reference |  |
| Tertile 2 | 1.84 (1.60, 2.12) | <0.0001 | 1.27 (1.09, 1.47) | 0.0016 | 1.04 (0.86, 1.25) | 0.7007 |
| Tertile 3 | 2.79 (2.45, 3.18) | <0.0001 | 1.85 (1.61, 2.13) | <0.0001 | 1.12 (0.88, 1.41) | 0.3690 |
| *P* for trend | <0.0001 |  | <0.0001 |  | 0.3575 |  |

In sensitivity analysis, the TyG index was converted from a continuous variable to a categorical variable (tertiles).

^1^OR : Odd ratio.

^2^95% CI : 95% confidence interval.

^3^Model 1 : No covariates were adjusted.

^4^Model 2 : Adjusted for age, sex, and race.

^5^Model 3 : Adjusted for sex, age, race, BMI, WC, education level, smoking, alcohol drinking, SBP, DBP, AST, ALT, serum uric acid, TC, LDL-C, HDL-C, serum total calcium, hypertension, and diabetes status.
