# Supplementary material for: Association between triglyceride-glucose index and chronic kidney disease: results from NHANES 1999–2020
Source: Int Urol Nephrol. 2024 Jun 10;56(11):3605–16. doi: 10.1007/s11255-024-04103-8 (PMC11464617; doi:10.1007/s11255-024-04103-8)
Supplement: Supplementary file 6 — Supplementary file6 (DOCX 13 KB) [file 11255_2024_4103_MOESM6_ESM.docx]

**Supplementary Table S3 |** Threshold effect analysis of TyG index on CVD using a two-piecewise linear regression model before and after adjustment of covariates.

| **CVD** | Before adjustment^3^ | After adjustment^4^ |
| --- | --- | --- |
| **Fitting by standard linear model** |  |  |
| OR^1^ (95%CI^2^) | 1.73 (1.62, 1.85) | 1.31 (1.07, 1.59) |
| *P-* value | < 0.0001 | 0.0074 |
| **Fitting by two-piecewise linear model** |  |  |
| Breakpoint (K) | 8.61 | 9.52 |
| OR1(< K ) | 3.43 (2.77, 4.24) | 1.19 (0.97, 1.48) |
|  | <0.0001 | 0.1019 |
| OR2(> K ) | 1.37 (1.24, 1.50) | 2.01 (1.31, 3.06) |
|  | < 0.0001 | 0.0012 |
| OR2 / OR1 | 0.40 (0.31, 0.52) | 1.68 (1.06, 2.66) |
|  | < 0.0001 | 0.0269 |
| Logarithmic likelihood ratio test *P*-value | <0.001 | 0.029 |

^1^OR: Odd ratio.

^2^95% CI: 95% confidence interval.

^3^Before adjustment: No adjustment.

^4^After adjustment: Adjusted for sex, age, race, BMI, WC, education level, smoking, alcohol drinking, SBP, DBP, AST, ALT, serum uric acid, TC, LDL-C, HDL-C, serum total calcium, hypertension, and diabetes status.
