# Supplementary material for: Association between triglyceride-glucose index and chronic kidney disease: results from NHANES 1999–2020
Source: Int Urol Nephrol. 2024 Jun 10;56(11):3605–16. doi: 10.1007/s11255-024-04103-8 (PMC11464617; doi:10.1007/s11255-024-04103-8)
Supplement: Supplementary file 7 — Supplementary file7 (DOCX 14 KB) [file 11255_2024_4103_MOESM7_ESM.docx]

**Supplementary Table S4 |** Comparison of AUC values between TyG index and other IR markers for predicting CVD.

| Test | AUC^1^ | 95%CI^2^ low | 95%CI upp | Best threshold | Specificity | Sensitivity | *P* for different in AUC |
| --- | --- | --- | --- | --- | --- | --- | --- |
| TyG index | 0.6242 | 0.6113 | 0.6371 | 8.5489 | 0.4906 | 0.6948 | Reference |
| LAP | 0.6213 | 0.6082 | 0.6344 | 31.7389 | 0.3814 | 0.7945 | 0.4213 |
| VAI | 0.5930 | 0.5791 | 0.6069 | 1.3917 | 0.4857 | 0.6581 | <0.0001 |
| TyG-BMI index | 0.5913 | 0.5777 | 0.6049 | 218.0641 | 0.3514 | 0.7827 | <0.0001 |

^1^AUC: area under the curve.

^2^95% CI: 95% confidence interval.
